# Tuberculous pleural effusion: integrating thoracic ultrasound and medical thoracoscopy in the diagnosis and management: a pictorial narrative review and an algorithm proposal

**DOI:** 10.1007/s40477-025-01094-9

**Published:** 2025-10-31

**Authors:** Riccardo Inchingolo, Lorenzo Carriera, Simone Ielo, Roberto Barone, Andrea Smargiassi, Roberto Lipsi, Stefano Baglioni, Raffaele Scala, Roberto Magri, Giampietro Marchetti

**Affiliations:** 1 UOC Pneumologia, Dipartimento Neuroscienze, Organi di Senso e Torace, Fondazione Policlinico Universitario A. Gemelli IRCCS, 00168 Rome, Italy; 2 Facoltà di Medicina e Chirurgia, Università Cattolica del Sacro Cuore, 00168 Rome, Italy; 3 Department of Pulmonology and Sub-Intensive Respiratory Unit, Ospedale Santa Maria Della Misericordia, Perugia, Italy; 4 Pulmonology and Respiratory Intensive Care Unit, Ospedale San Donato, USL Toscana Sud-Est, Arezzo, Italy; 5 Pulmonology Department, ASST Spedali Civili, Brescia, Italy

**Keywords:** Tuberculosis, Tuberculous pleural effusion, Thoracic ultrasound, Medical thoracoscopy, Internal Mammary Lymph Node

## Abstract

Tuberculous pleural effusion (TPE) is currently the most common form of extrapulmonary tuberculosis and remains a significant cause of pleural disease worldwide, particularly in endemic regions. It often presents with non-specific clinical symptoms, such as fever, chest pain, cough and weight loss, thereby complicating the diagnostic process. This narrative review provides an expert comprehensive clinical practice overview covering the following issues correlated with diagnosis and management of TPE: 1- epidemiology, clinical presentation and underlying pathophysiology; 2- limitations of conventional diagnostic procedures on pleural fluid; 3- usefulness of thoracic ultrasound (TUS) in the stepwise pathway 4- role of thoracoscopy as a golden diagnostic tool, with the proposal of a step by step algorithm. A large iconography enriches the review from the educational point of view by presenting a pictorial series of characteristic sonographic and thoracoscopic findings.

## Introduction

Tuberculous pleural effusion (TPE) is currently the most common form of extrapulmonary tuberculosis (TB) [1]. It is more frequently observed in endemic areas, especially in individuals co-infected with HIV, consistent with epidemiological data indicating a higher risk of TB in immunocompromised patients [2]. TPE may result from a primary infection acquired within the preceding 6–12 weeks or, more commonly, from reactivation of latent TB, triggered by diseases (ie. solid haematologic malignancies) or systemic treatments (prolonged steroids, antiblastic and biologic therapies) associated with depressed cell-mediated immune response [3]. In industrialized countries, however, it typically represents a post-primary manifestation, occurring at a later mean age compared to that reported in other regions [4]. Despite advances in microbiological and immunological diagnostics, the identification of pleural TB continues to pose a challenge due to the pauci-bacillary nature of the disease and the often non-specific clinical and radiographic presentation. Medical thoracoscopy (MT), a minimally invasive endoscopic procedure, plays a pivotal role in the evaluation of undiagnosed exudative pleural effusions, offering both direct visualization of the pleural space and the opportunity to obtain targeted biopsies under direct vision. Its diagnostic yield in pleural tuberculosis is significantly higher than that of closed pleural biopsy and other non-invasive methods, making it a corner-stone in the modern diagnostic algorithm [5]. In the last decades, thoracic ultrasound (TUS) has changed the clinically practice paradigm for clinicians to approach suspected or demonstrated pleural diseases, by quickly selecting those patients who require either conventional and/or advances diagnostic procedures such as MT to get the correct and rapid diagnosis. This is the case of TPE, given to the challenges often faced by clinicians in detecting this disease outside the typical pulmonary TB scenarios. This pictorial review is a narrative expert’s comprehensive report on TPE aiming to focus on the following key points: 1- epidemiology, clinical presentation and underlying pathophysiology; 2- limitations of conventional diagnostic procedures on pleural fluid; 3- usefulness of TUS in the diagnostic pathway together with the potential value of the Internal Mammary Lymph Node detection 4- role of thoracoscopy as a diagnostic tool, with the proposal of a step-by-step algorithm. This reviews would like also to add an educational and iconographic value by showing a large series of selected images and clinical examples: key ultrasonographic and thoracoscopic findings associated with tuberculous pleuritis, ranging from classical macroscopic features, such as pleural nodules, adhesions, and caseating necrosis—with the correlated histopathological patterns.

## Pathogenesis of TPE

The underlying pathogenic mechanism involves a T-cell–mediated hypersensitivity reaction following the entry of mycobacterial antigens into the pleural space, often after the rupture of subpleural caseating granulomas. The presence of these antigens triggers an inflammatory response, leading to increased capillary permeability and osmotic transudation of proteins and fluid into the pleural cavity. The ensuing inflammation, initially dominated by neutrophils and subsequently by T lymphocytes, impairs lymphatic drainage by obstructing pleural lymphatic channels, thereby slowing fluid reabsorption. The robust infiltration of Th1-type T cells creates a localized immune microenvironment, referred to as compartmentalization, which explains both the paucibacillary nature of TPE and the low yield of mycobacterial cultures obtained from pleural fluid [1, 6]. In some cases, as the disease progresses, the inflammatory profile may shift from a lymphocyte-predominant to a neutrophil-predominant response [7]. These presentations are often associated with loculated effusions or the development of complicated effusions and frank empyemas, which are likely to achieve higher rates of culture positivity for Mycobacterium tuberculosis [8].

## TPE: pleural fluid characteristics and diagnostic markers

TPE typically appear pale yellow and are classified as exudates. Cytological analysis characteristically reveals lymphocytosis, with lymphocytes accounting for more than 50% of the total nucleated cells, and in most cases, exceeding 75%. It is worth noting, however, that TPE is typically characterized by a high neutrophil count during the very early stages of disease or in later stages consistent with empyema [1, 9]. Lymphocytes become predominant in the phases following the initial immune response, usually after approximately two weeks from the onset of the hypersensitivity reaction [1, 3]. When uncomplicated, the pleural fluid usually has a pH above 7.3. Consistent with the features of exudates, TPE fluid shows elevated lactate dehydrogenase (LDH) levels compared to serum and a protein concentration that may exceed 5 g/dL. The presence of mesothelial cells is rare, although it is more frequently observed in patients co-infected with HIV [10]. As previously discussed, pleural fluid cultures are infrequently positive for Myco-bacterium tuberculosis, which limits their diagnostic utility in suspected tuberculous pleural effusion (TPE). In this context, adenosine deaminase (ADA), an enzyme produced and released by activated T lymphocytes, represents a low-cost, widely accessible, and clinically valuable biomarker. ADA measurement in pleural fluid is considered both highly sensitive and specific for TPE, particularly when levels exceed a diagnostic threshold of 40 U/L [11]. A lymphocyte-predominant exudate combined with elevated ADA levels is highly suggestive of tuberculosis in high-prevalence settings. On the contrary, in low-prevalence regions, the absence of lymphocyte predominance and elevated ADA levels makes a tuberculous etiology unlikely, and pleural biopsy should be considered to confirm the diagnosis. Importantly, ADA levels are not significantly influenced by the patient’s HIV status [11, 12], nor are they merely a reflection of the absolute lymphocyte count in the pleural fluid, which underscores the robustness of this marker across different clinical settings [13]. Nonetheless, elevated ADA levels may also be observed in non-tuberculous empyemas, potentially leading to false-positive interpretations [14]. In such cases, diagnostic specificity can be significantly enhanced by assessing the pleural fluid lymphocyte-to-neutrophil ratio, with values greater than 0.75 being more strongly suggestive of TPE [12]. Another potential diagnostic tool that has been recently proposed is the measurement of interferon-gamma levels in pleural fluid [15]. This cytokine, released by activated CD4 + T cells, plays a key role in the host immune response to Mycobacterium tuberculosis, increasing the mycobactericidal activity of macrophages. Interferon-γ release assays, such as QuantiFERON-TB Gold, have been developed for diagnosis of latent tuberculosis, measuring interferon-γ release by sensitized T cells from peripheral blood in response to Mycobacterium tuberculosis-specific antigens. Despite being good at identifying patients who have been infected with Mycobacterium tuberculosis, recent meta-analyses indicated that the performances of blood interferon-γ release assays were unsatisfying to identify patients with TPE [16, 17]. Some authors have then proposed the use of Interferon Gamma Release Assay (IGRA) on pleural fluid as a potential earlier diagnostic tool for TPE, aiming to capture the local cellular immune activity [18]. In fact, the immune response in tuberculous pleuritis is compartmentalized, with a pre-dominance of CD4 + lymphocytes and elevated interferon-gamma levels in the pleural fluid, reflecting local Th1-mediated macrophage activation and mycobacterial control. Unfortunately, when applied to pleural fluid, IGRAs remain prone to false-positive results, particularly in individuals with latent TB or with HIV coinfection [18]. This is likely due to the passive migration of circulating presensitized T cells into pleural effusions of non-tuberculous origin, which reduces test specificity. These limitations are especially relevant in endemic areas, where latent infection is common, and they significantly restrict the usefulness of IGRAs for reliably diagnosing tuberculous pleuritis [19]. In consideration also of the absence of standardized cut-off values, higher costs, and a diagnostic yield that does not substantially surpass that of ADA measurement, current evidence remains insufficient to support their routine use in clinical practice [3, 20]. Therefore, ADA remains the preferred initial test in the evaluation of suspected TPE in most clinical settings. Finally, molecular tests based on nucleic acid amplification can identify Mycobacterium tuberculosis directly in pleural fluid within a short timeframe. However, despite their high specificity, their diagnostic sensitivity remains inconsistent and generally low; for this reason, their routine use in tuberculous pleuritis is not currently recommended [21]. In addition to the reported limitations in the diagnostic reliability of these bio-molecular markers, the clinicians should take in mind the therapeutic drawbacks correlated with the unavailability of cultures of Mycobacterium tuberculosis and their sensitivity to the target antibiotic drugs.

## Clinical presentation

TPE may present acutely, particularly in younger, immunocompetent individuals, or follow a more subacute course [3]. The most common clinical manifestations include pleuritic chest pain, nonproductive cough, fever, night sweats, dyspnoea, and weight loss [1]. The effusion is typically unilateral and can vary widely in size. Patients with TPE are generally younger than those with pulmonary tuberculosis; however, in industrialized countries, tuberculous pleuritis more commonly reflects reactivation disease, and the average patient age tends to be higher [3]. Although some individuals experience only mild symptoms, up to 65% of these cases may progress to active pulmonary tuberculosis within two years, underscoring the importance of early diagnosis and prompt initiation of therapy [1]. Patients who are HIV-positive are more likely to present with fever, cough, weight loss and systemic symptoms compared to non-HIV-positive patients [22].

## Ultrasound characteristics of TPE

**Fig. 1:**
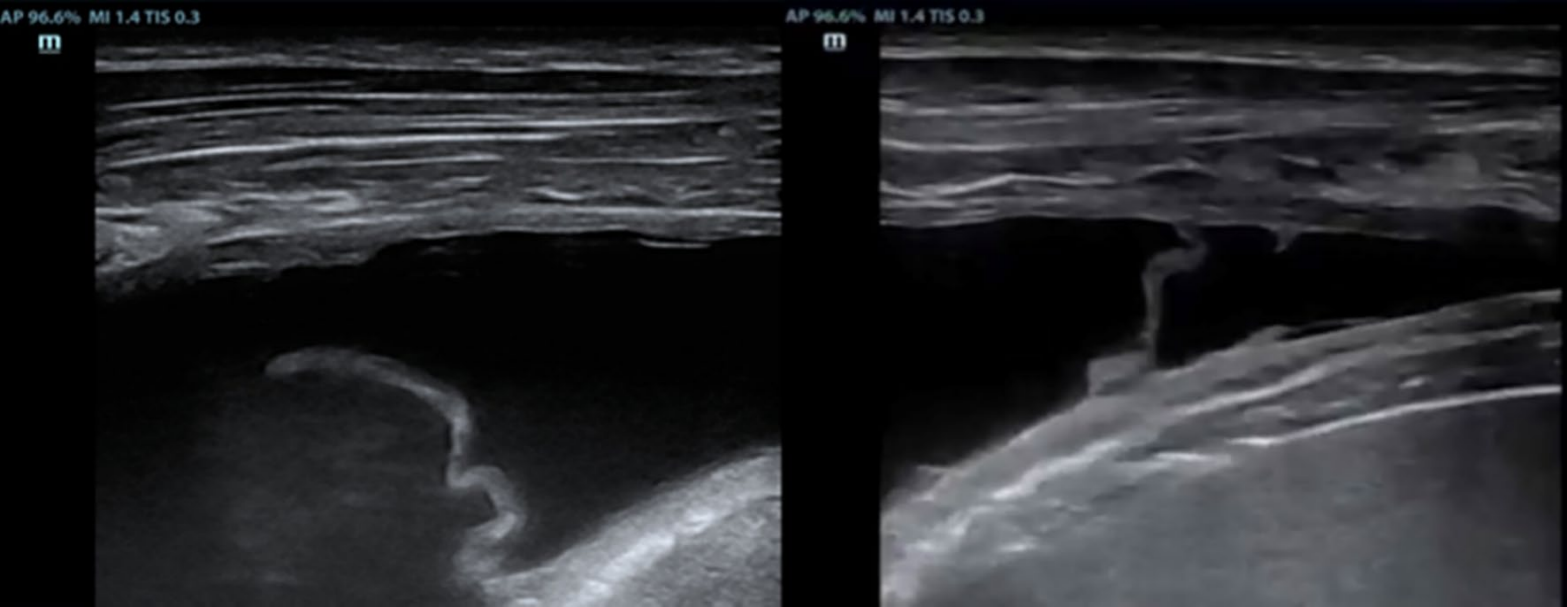
TUS showing a typical TPE. The images reveal an anechoic, free flowing pleural fluid. Thin, mobile fibrinous strand are visualized within the pleural space. Such sonographic findings, while not pathognomonic, are highly suggestive of a tuberculous etiology in the appropriate clinical context

Diagnostic work-up of TPE is usually challenging. In recent years, the growing use of thoracic ultrasound (TUS) in respiratory medicine has transformed the diagnostic approach [23]. Initially employed to confirm the presence of pleural effusion and quantify its extent, TUS has more recently been proposed as a valuable tool for identifying specific features of the effusion and guiding the diagnostic work-up of these patients [9]. Most commonly, TUS reveals an anechoic, free-flowing effusion, often accompanied by small, mobile, and immature fibrin strands (Fig. 1).

**Fig. 2:**
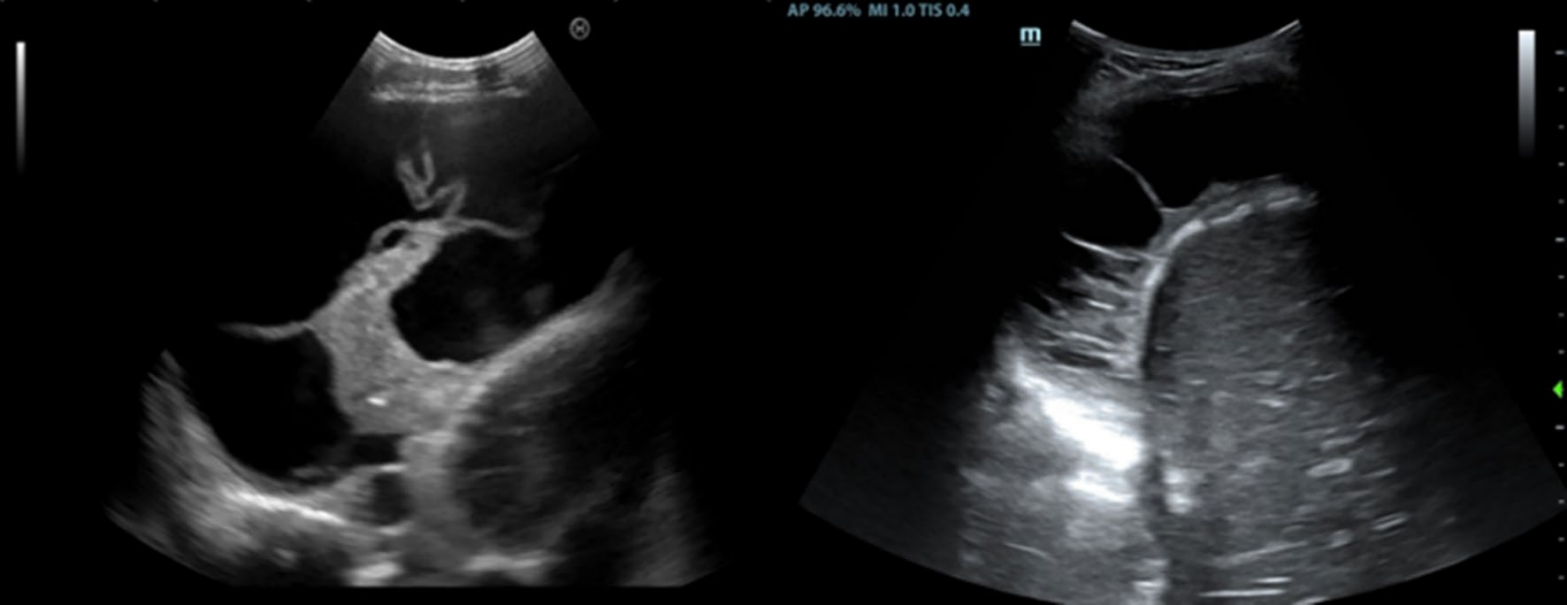
**a** The pleural space contains multiple thin, filamentous fibrin strands. These delicate septations, that are highly mobile and represent an early stage or organization within the effusion, may form large loculated fluid pockets. **b** A more complex pleural effusion is observed, characterized by thick echogenic septations. These findings reflect a more advance stage of the disease, with extensive organization of the pleural fluid

The fibrin strands, being thin and filamentous, can occasionally create large loculated pockets (Fig. 2a). Less commonly, the effusion appears complex, with evidence of thick septations and loculations (Fig. 2b).

**Fig. 3:**
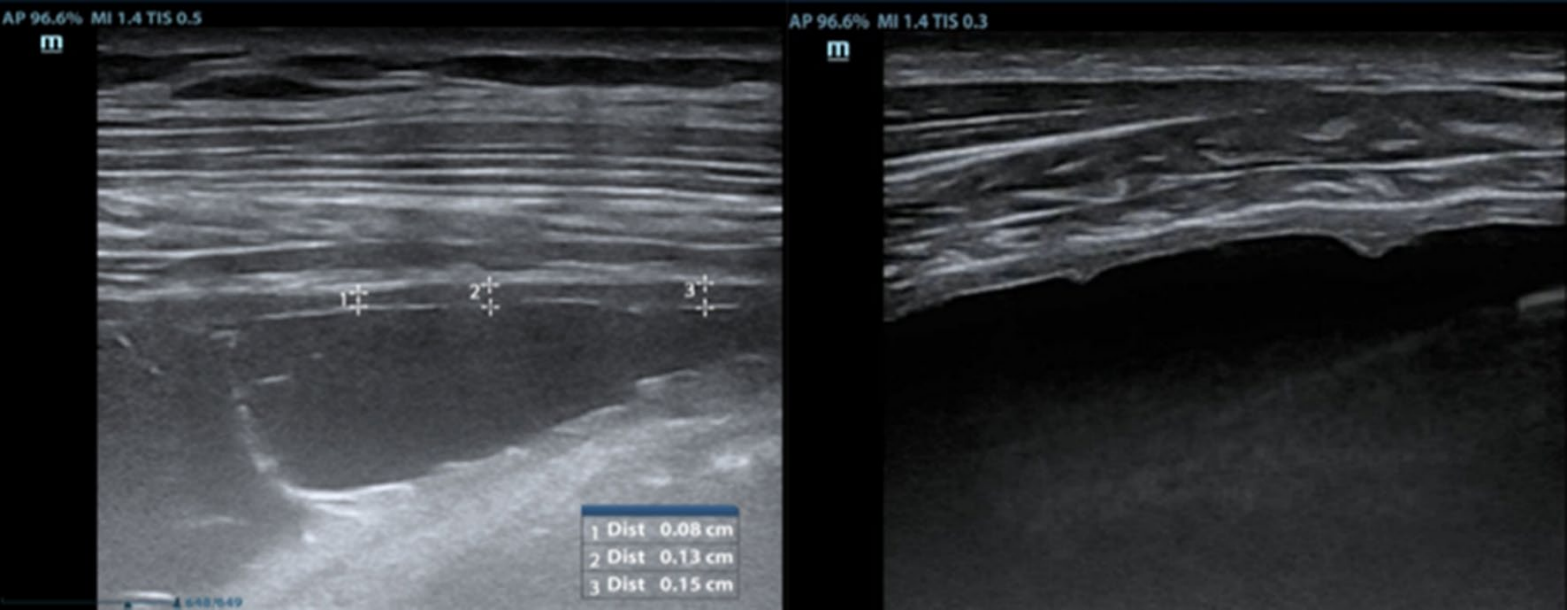
**a** Diffuse pleural thickening, indicative of chronic inflammatory involvement of the pleura. **b** Pleural thickening is accompanied by multiple hypoechoic nodular formations adherent to the pleural surface, markers of active granulomatous inflammation

Additional ultrasonographic findings features that may be associated with TPE include pleural thickening (Fig. 3a) without or with US visible superimposed nodular formations, indicative of active inflammation (Fig. 3b).

Finally, TUS plays a key role not only in the diagnosis but also in the therapeutic management of TPE. It allows for serial monitoring of the effusion over time, facilitates the early detection of complications and supports clinical decision-making, including the initiation of adjunctive therapies when indicated.

## TUS evaluation of Internal Mammary Lymph Node: a sentinel sign in TPE

**Fig. 4:**
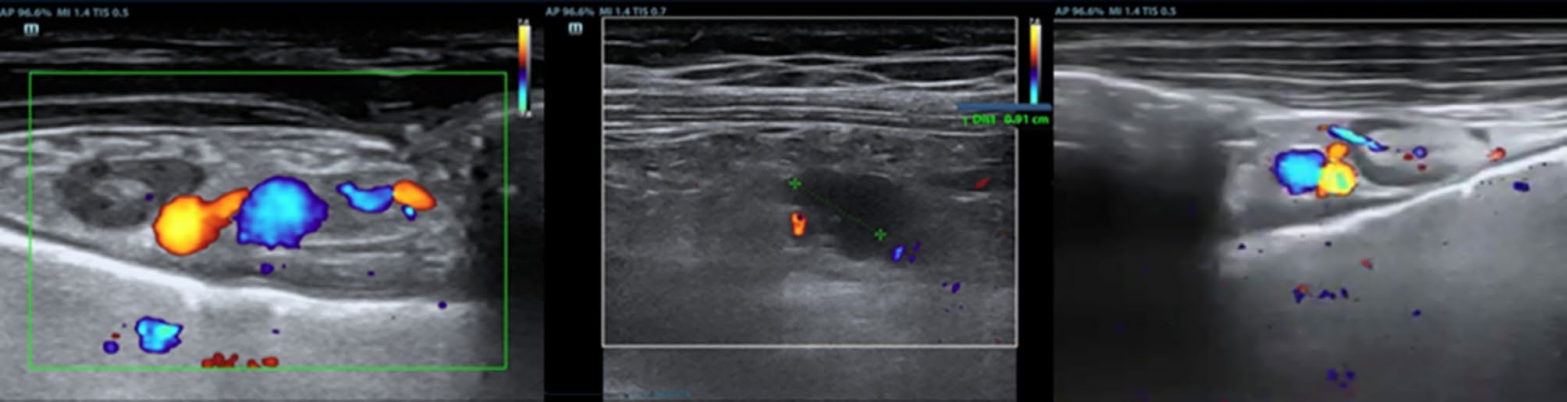
The IMLN is more frequently visualized at the second and third intercostal space on the parasternal line, lateral to the internal mammary vessels (**a**), between the artery and the vein (**b**) or medial (**c**)

**Fig. 5:**
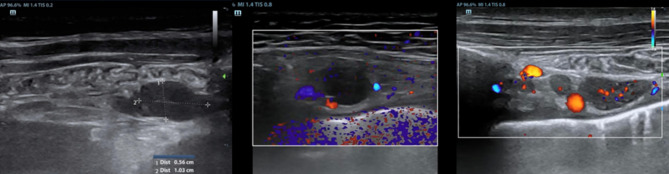
**a** An IMLN can be considered as pathological when the short diameter is > 5 mm. **b** In TPE, it often exceeds 10 mm. **c** Sometimes, even multiple lymph nodes can be detected

A recently proposed application of TUS is the evaluation of ipsilateral Internal Mammary Lymph Node (IMLN). Its visualization on the same side as the pleural effusion may serve as a sentinel sign of tuberculous pleurisy, aiding in early diagnosis and guiding the diagnostic approach [24]. In a recent multicenter Italian study [25], pathological IMLN findings were in fact associated with an increased pre-test probability of tuberculous etiology. This is especially true in younger patients and when the lymph nodes exceed 10 mm in diameter, suggesting the need for more invasive diagnostic procedures. Conversely, the absence of IMLN involvement, particularly in older individuals, can nearly exclude a tuberculous origin. TUS is easy and rapid to perform when there is adequate operator expertise and can be performed bedside or in outpatient setting. Therefore, given its diagnostic value and ease of use, TUS with IMLN involvement evaluation should be considered the first-line approach in patients with suspected pleural tuberculosis. (Figs. 4 and 5).

## Tissue is the issue

**Fig. 6:**
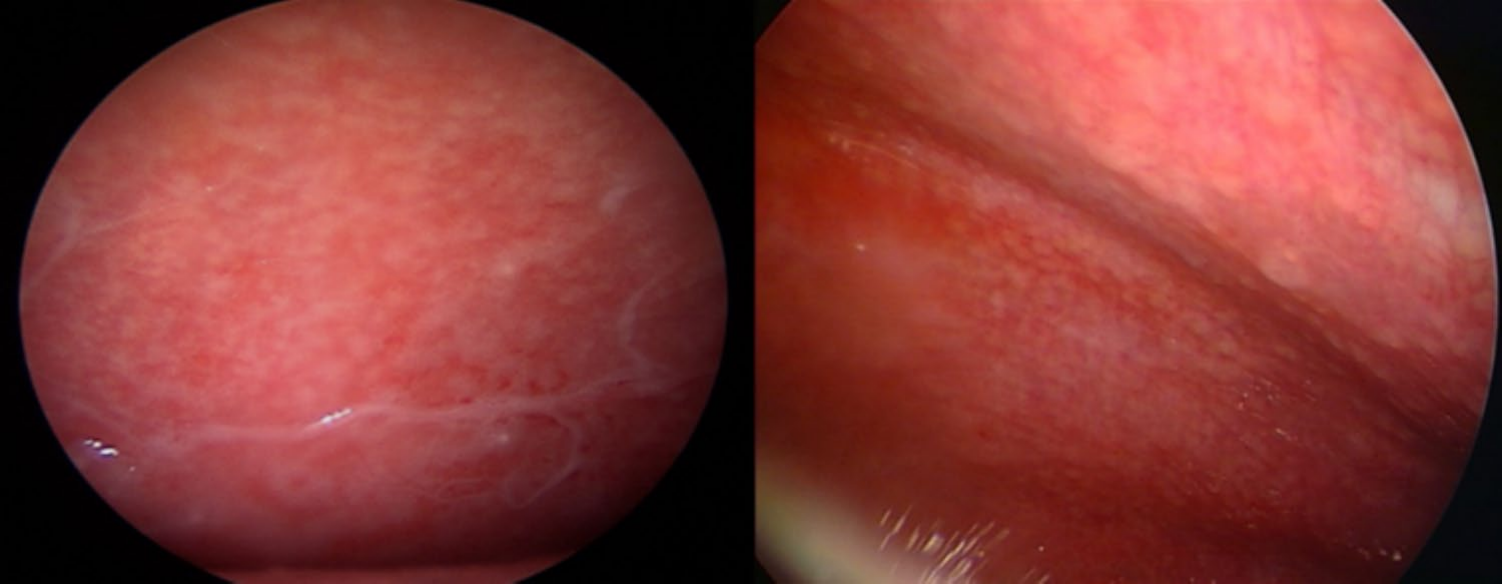
“Strawberry-like” appearance of parietal pleura. Numerous diffuse pale micronodules distributed across a markedly hyperemic surface. This pattern reflects active granulomatous inflammation

**Fig. 7:**
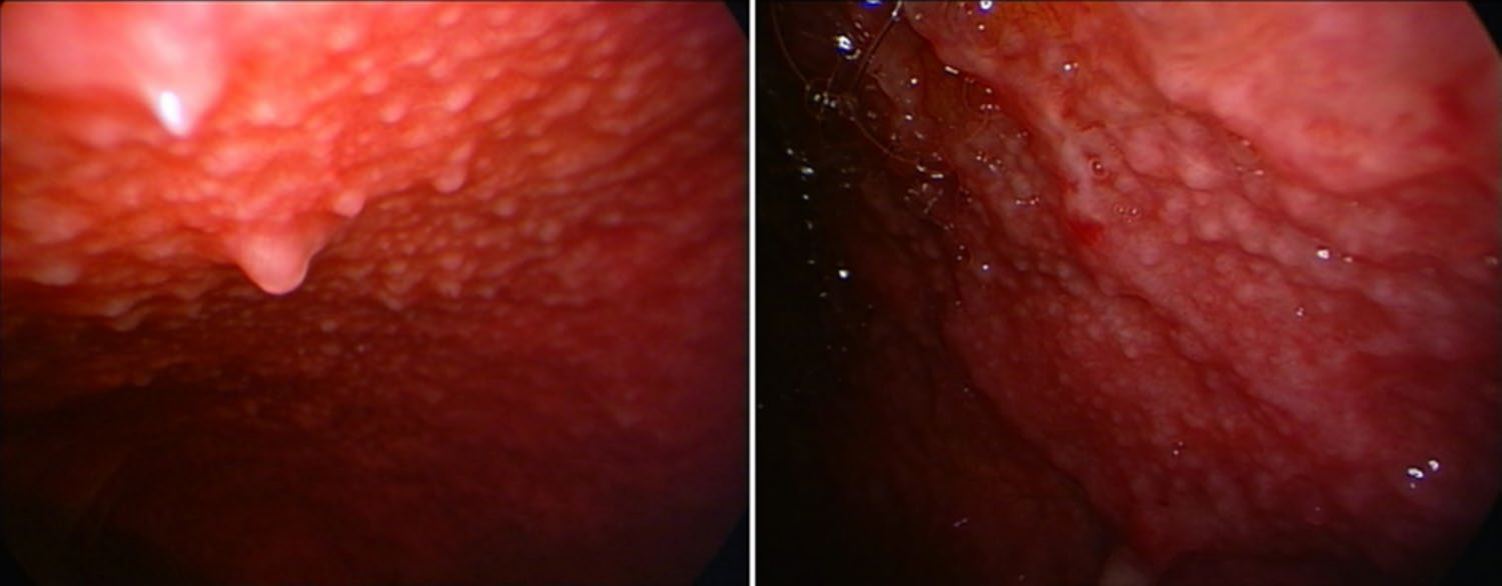
“Sago-like” nodules on parietal pleura. Multiple white nodules resembling sago pearls protruding from the surface. This macroscopic presentation is highly specific and correlates with underlying caseating granulomas on histopathology

Ultrasound has become also a valuable tool in guiding interventional procedures. This has been particularly evident in the case of MT, where ultrasound guidance has significantly enhanced procedural safety and accuracy and reduced the risk of complications [26]. For the past 50 years, the most common method for diagnosing tb pleural effusion has been blind needle biopsy of the pleura [3]. Specifically in the context of tuberculous pleuritis, MT has emerged as an effective and safe diagnostic tool, with a high diagnostic yield [5, 27]. It has been recently showed that MT performed under analgosedation by a pulmonologist team is a safe and well-tolerated procedure with a diagnostic yield similar to that obtained in VATS both for neoplastic and non-malignant pleural effusion [28]. MT allows direct visualization of the pleural cavity and the collection of pleural fluid for di-agnostic analysis, including direct microscopic examination with smear for acid-fast bacilli (AFB) on Ziehl Neelsen stain, culture, and molecular testing. Fibrinous material collected during thoracoscopy can be tested using the same diagnostic techniques, possibly leading to successful isolation of Mycobacterium tuberculosis. However, TPE develops as a result of delayed hypersensitivity reaction to mycobacterial antigens. Therefore, the bacterial load in the pleural space is typically low, which explains why direct microscopy and culture of the fluid are often negative (the diagnostic yield is 5–10% for pleural fluid AFB smear and 30–50% for culture) [29]. This makes pleural biopsy the most reliable tool for achieving a definitive diagnosis [30], which is based on the detection of Mycobacterium tuberculosis in pleural fluid or biopsy specimens, or on histopathological evidence of epithelioid cell granulomas and/or caseating granulomas. Pleural tissue specimens obtained during thoracoscopy can be used for microbiological and histological analyses. They should be sent for AFB culture, Xpert MTB/RIF testing [31] and histopathological examination looking for the presence of granulomas. These steps may be of help for clinicians to drive the appropriate antimicobacterial combined therapy on the basis of antibiogram. During MT performed in cases of suspected TPE, several distinct endoscopic patterns can be observed. The most common abnormalities include diffuse pale micronodules on a hyperemic background, which give the pleural surface a “strawberry-like” appearance (Fig. 6), and “sago-like” nodules, so called due to its resemblance to sago pearls (Fig. 7). This pattern is considered highly specific for tuberculous involvement [32].

**Fig. 8:**
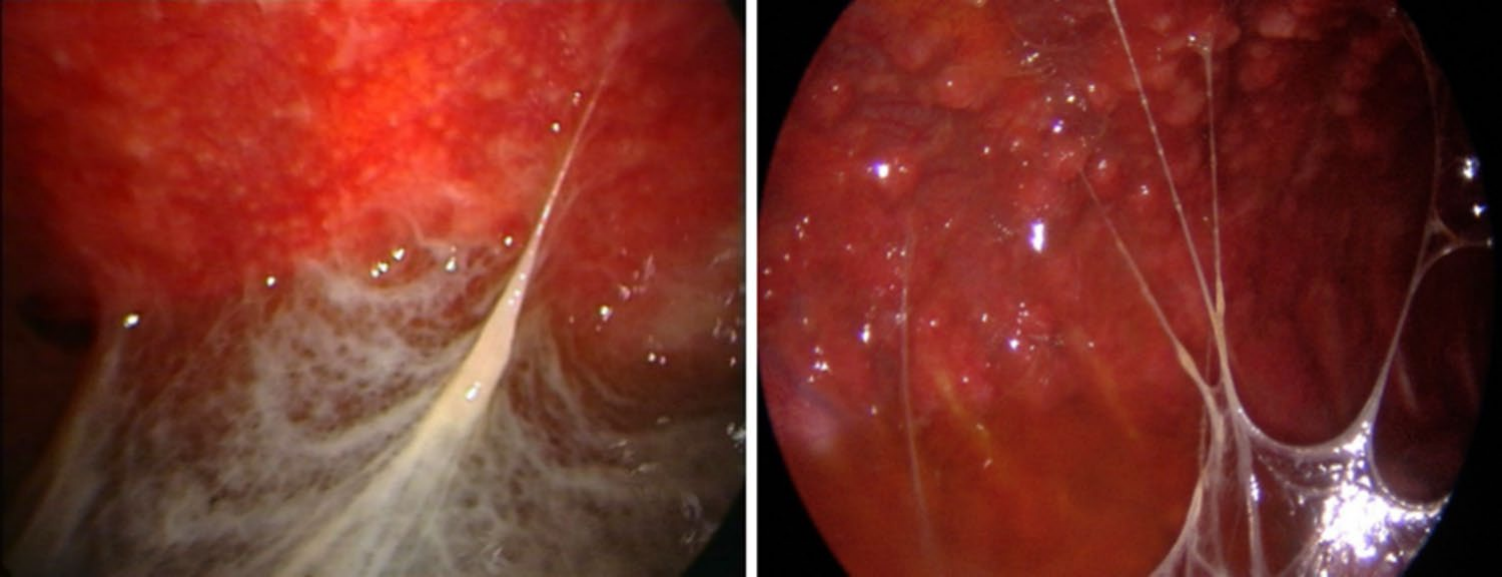
Delicate fibrin brands visualized within the pleural cavity, indicative of an early-phase inflammatory response. Fibrinous material obtained during thoracoscopy can frequently yield positive results for Mycobacterium tuberculosis

In the appropriate clinical context, this endoscopic appearance alone may justify the early initiation of anti-tuberculosis treatment while awaiting histological and microbiological confirmation. In fact, a strong association between the presence of sago-like nod-ules and detection of Mycobacterium tuberculosis on culture and Xpert MTB/Rif assay of pleural biopsies has been described. Interestingly, also granulomas are more frequently found in pleural biopsies from patients with this nodular pattern [27]. Thoracoscopy may reveal delicate and recently formed fibrin bands, which are often associated with the early stages of pleural inflammation (Fig. 8).

**Fig. 9:**
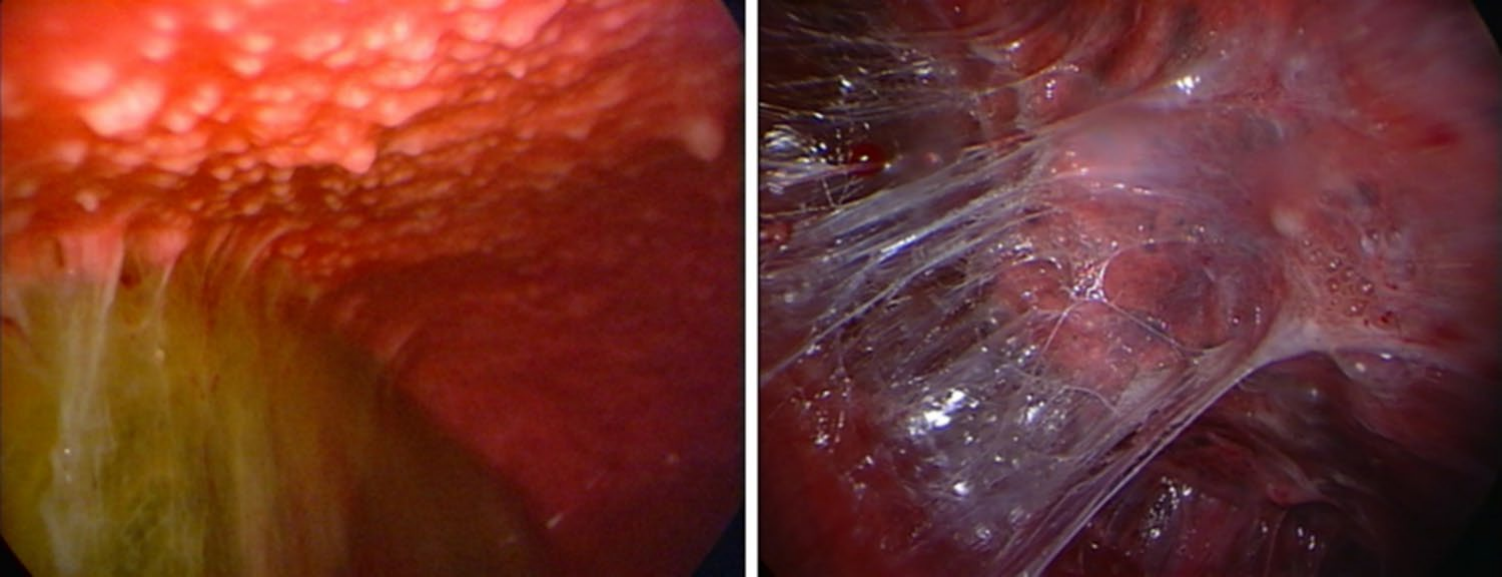
These fibrous bridges represent chronic pleural involvement, resulting from the evolution of previously delicate fibrin bands

**Fig. 10:**
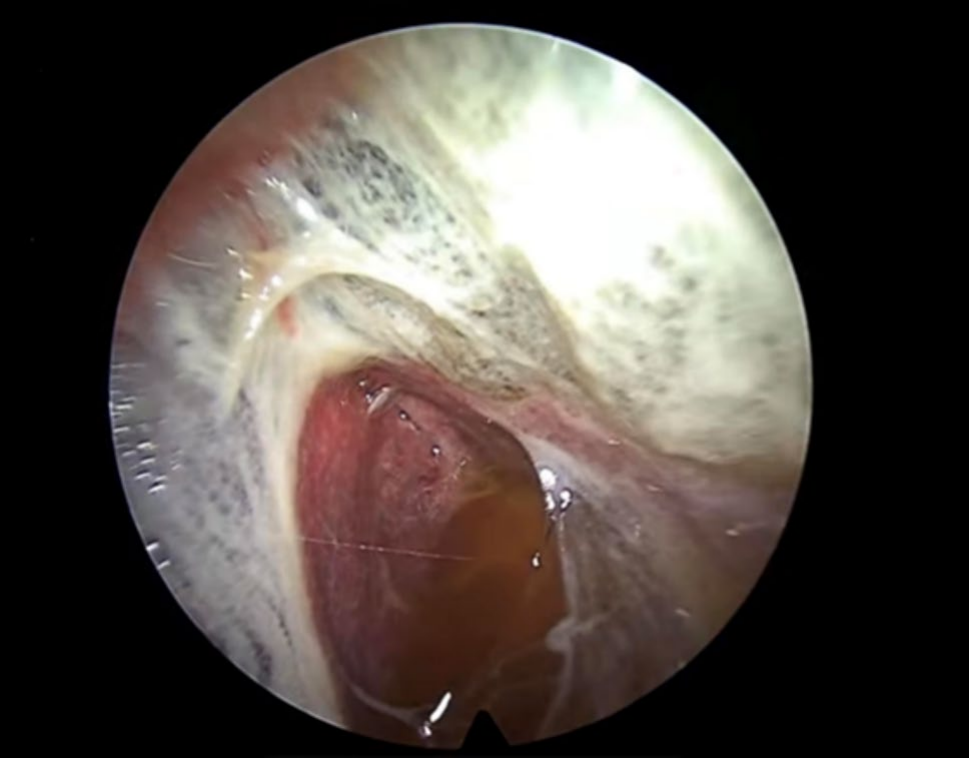
Formation of complex septated pleural cavity. Multiple dense fibrinous septa create loculated compartments significantly complicating fluid drainage. These septa can be lysed during thoracoscopy using a spoon shaped forceps. The intrapleural administration of fibrinolytic agents may further assist in accelerating the resolution process

When long-standing, these fibrin bands can sometimes result in visible macroscopic adhesions (Fig. 9), or even create complex and septated pleural cavities (Fig. 10).

In these cases, the use of intrapleural fibrinolytic therapy (IPFT), particularly urokinase, alongside antituberculous therapy, has proven effective in promoting faster drainage of the effusion, reducing residual pleural thickening, and improving respiratory function [33]. Even if still debated in TPE, this approach mirrors what is commonly done in the treatment of complicated parapneumonic effusions and empyemas, where fibrinolytics are routinely employed to enhance fluid resolution and prevent long-term sequelae [34]. If multiple and substantial biopsies have been performed during MT, and the complex effusion has been mechanically debrided using a spoon forceps, it is advisable to wait 24 h before administering IPFT into the pleural cavity.

**Fig. 11:**
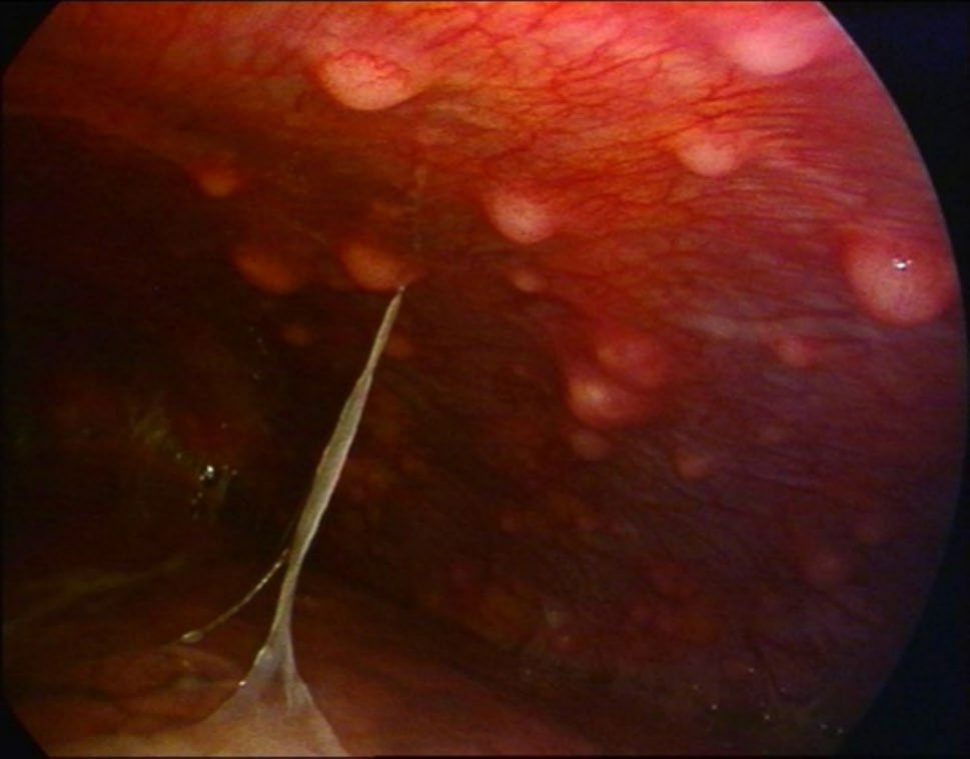
Pustules (pus-filled nodules) can be observed on the parietal pleura

Pleural pustules (Fig. 11) have also been described in the literature. When present, they exhibit a high positive predictive value for tuberculous pleural effusion, and biopsies from these lesions are more likely to yield a positive microbiological diagnosis [35].

**Fig. 12:**
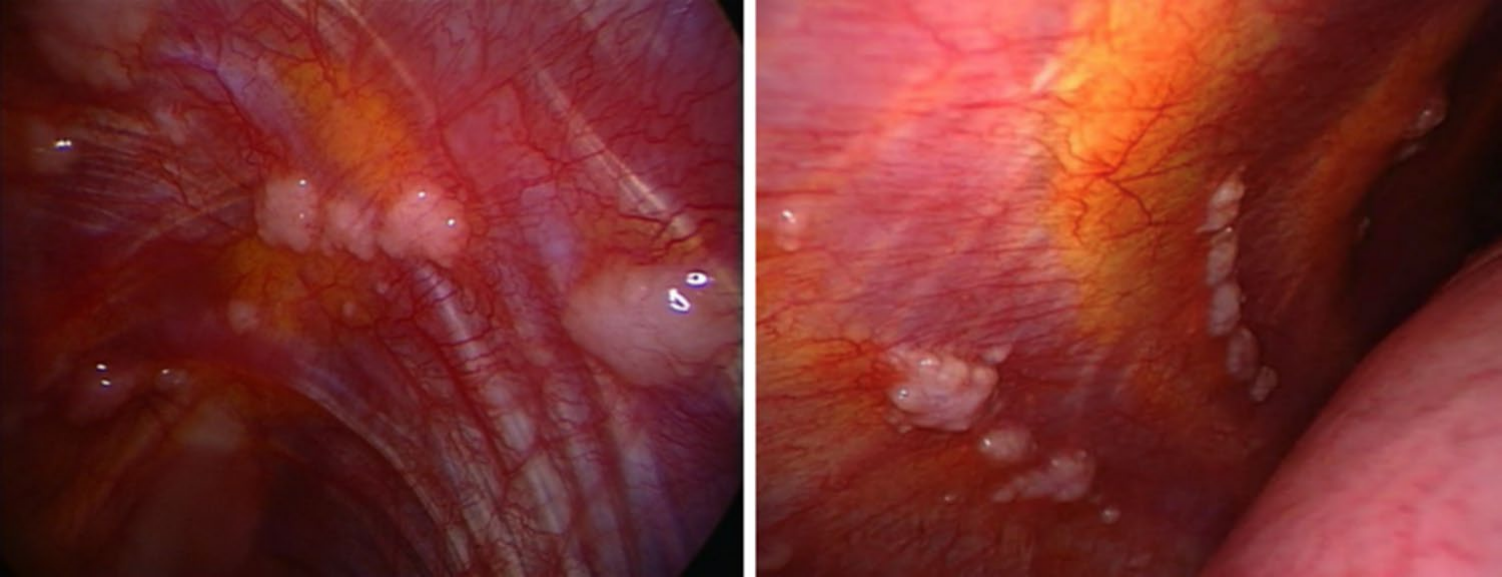
Atypical endoscopic manifestation of tuberculous pleurisy. The pleural surface is studded with multiple whitish, firm, mulberry-like nodular lesions

The high rate of immigration in countries with low tuberculosis prevalence has led to an increasingly frequent observation of atypical endoscopic patterns of tuberculous pleurisy. These presentations differ from the classic “strawberry-like” pleura and may closely mimic malignant pleural involvement, thus posing significant challenges in the differential diagnosis. For instance, Fig. 12 shows whitish, firm, mulberry-like lesions.

## Discussion

**Fig. 13:**
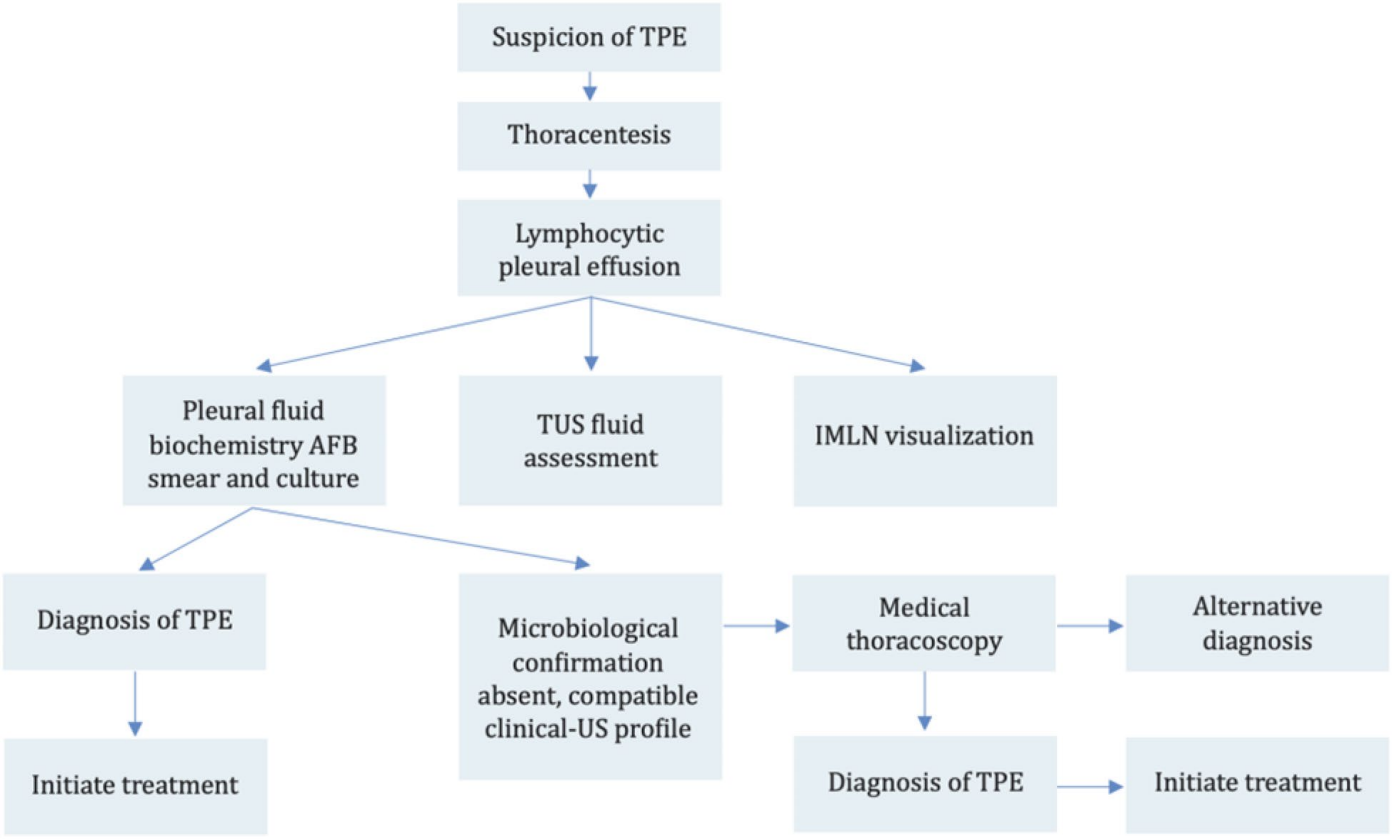
A diagnostic algorithm for the evaluation of patients presenting with clinical and radiological features suggestive of TPE

With this narrative review, we would like to provide a comprehensive and practical- expert-based report on sinergistic role of the integrated sequential application of TUS and MP within the diagnosis and management of TPE highlighting the limitations of the conventional bio-molecular markers on pleural fluid. Integrating TUS into the routine diagnostic workup of TPE can significantly enhance diagnostic accuracy and accelerate clinical management, particularly in high-burden or resource-limited settings. In patients from TB-endemic countries, who therefore have a high pre-test probability and present with a lymphocytic exudate and elevated ADA levels, the identification of typical ultrasound findings may serve as a valuable adjunct and further supports the decision to initiate treatment. Although MT offers the highest diagnostic yield for both pleural malignancy and TB and is considered the gold standard, it may not be easily accessible in low-resource settings. In these contexts, repeating an ultrasound-guided thoracentesis may be a reasonable alternative before proceeding to pleural biopsy. In low-incidence countries, where most TPE cases are associated with immigration, the identification of a positive IMLN on ultrasound, especially in younger patients with a compatible clinical picture, has demonstrated a high positive predictive value and can be virtually diagnostic of TPE. In such cases, MT enables diagnostic confirmation with near 100% sensitivity. Decision-making in the diagnostic workup of TPE involves a stepwise approach integrating clinical, laboratory, and imaging data. A practical diagnostic approach is presented in Fig. 13.

When TPE is suspected, thoracentesis is performed as the initial step. The identification of a lymphocytic pleural effusion prompts further evaluation with pleural fluid bio-chemistry, AFB smear and culture, TUS and assessment of IMLN. If microbiological confirmation is achieved, a diagnosis of TPE is established, and treatment can be initiated. However, as previously discussed, direct microscopy and culture of the fluid are often negative (the diagnostic yield is 5–10% for pleural fluid AFB smear and 30–50% for culture) [29]. TUS therefore plays a pivotal role in assessing the pre- invasive test probability of TB by identifying characteristic patterns. If clinical and sonographic features are strongly suggestive of TPE, further evaluation with MT is warranted. This procedure can confirm the diagnosis or guide toward an alternative. Recently, also ultrasound-guided pleural biopsy has been proposed as a technique to obtain histological samples. Although its effectiveness and safety still need to be validated in larger studies, early results are promising: the diagnostic yield appears comparable to that of MT, with the advantage of being less invasive in patients with comorbidities and adequate pleural thickness. Thoracoscopic biopsies, however, remain preferable in cases of massive pleural effusion or when adhesiolysis is required [36].

The main limitation of this paper is the non-systematic design of the review that does not follow the criteria of the statistical meta-analysis based on randomized controlled studies within the context of a topic such as TPE diagnosis – when evidenced based data are scanty. However, our aim was to provide a narrative review based on expert experience coming from the clinical everyday practice. We believe that the strength of this review is overall its educational value for what concern the presentation of a large series of the ultrasonographic and thoracoscopic pictures taken by the real life practice depicting various findings of tuberculous pleuritis, (ie. pleural nodules, adhesions, and caseating necrosis) which are strongly correlated with specific histopathological patterns. Moreover, the included proposed algorithm may be of help for clinicians to better select the cases of suspected TPE to be submitted to MT to avoid delayed time for diagnosis and treatment, therefore preventing the potential uncontrolled spreading of the infections.

## Conclusions

Tuberculosis remains a significant challenge for modern medicine and is the world’s leading cause of death from a single infectious agent [37]. In recent decades, the emergence of multi-resistant strains (MDR-TB and XDR-TB) has complicated disease control and highlighted the need for new diagnostic and therapeutic strategies [38]. In this context, early diagnosis using molecular techniques, advanced imaging and chest ultrasound has become increasingly important, particularly for extrapulmonary manifestations such as TPE, which often has an unclear clinical presentation [39]. The treatment of TPE is directed toward preventing the progression to active tuberculosis, alleviating the patient’s symptoms, and minimizing the risk of complications, including fibrothorax development [3]. MT has emerged as the gold standard for the diagnosis of TPE. Its ability to directly visualize the pleural cavity revealing characteristic pathological findings and obtain targeted tissue biopsies offers significant advantages over traditional diagnostics methods. The integration of TUS and MT into routine clinical practice could significantly reduce the diagnostic delay and improve therapeutic management of TPE, thereby reducing associated morbidity and mortality.

## Data Availability

Not applicable.
